# Analysis of Altered MicroRNA Expression Profiles in Proximal Renal Tubular Cells in Response to Calcium Oxalate Monohydrate Crystal Adhesion: Implications for Kidney Stone Disease

**DOI:** 10.1371/journal.pone.0101306

**Published:** 2014-07-01

**Authors:** Bohan Wang, Bolin Wu, Jun Liu, Weimin Yao, Ding Xia, Lu Li, Zhiqiang Chen, Zhangqun Ye, Xiao Yu

**Affiliations:** 1 Department of Urinary Surgery, Tongji Hospital of Huazhong University of Science and Technology, Wuhan, Hubei Province, China; 2 Department of Urinary Surgery, Affiliated Second Hospital of Zhejiang University, Hangzhou, Zhejiang Province, China; 3 Department of Urinary Surgery, Suining Centre Hospital, Suining, Sichuan Province, China; The University of Manchester, United Kingdom

## Abstract

**Background:**

Calcium oxalate monohydrate (COM) is the major crystalline component in kidney stones and its adhesion to renal tubular cells leads to tubular injury. However, COM-induced toxic effects in renal tubular cells remain ambiguous. MicroRNAs (miRNAs) play an important role in gene regulation at the posttranscriptional levels.

**Objective:**

The present study aimed to assess the potential changes in microRNAs of proximal renal tubular cells in response to the adhesion of calcium oxalate monohydrate (COM) crystals.

**Methodology:**

Lactate dehydrogenase (LDH) activity and DAPI staining were used to measure the toxic effects of HK-2 cells exposed to COM crystals. MicroRNA microarray and mRNA microarray were applied to evaluate the expression of HK-2 cells exposed to COM crystals. Quantitative real-time PCR (qRT-PCR) technology was used to validate the microarray results. Target prediction, Gene Ontology (GO) analysis and pathway analysis were applied to predict the potential roles of microRNAs in biological processes.

**Principal Findings:**

Our study showed that COM crystals significantly altered the global expression profile of miRNAs in vitro. After 24 h treatment with a dose (1 mmol/L), 25 miRNAs were differentially expressed with a more than 1.5-fold change, of these miRNAs, 16 were up-regulated and 9 were down-regulated. A majority of these differentially expressed miRNAs were associated with cell death, mitochondrion and metabolic process. Target prediction and GO analysis suggested that these differentially expressed miRNAs potentially targeted many genes which were related to apoptosis, regulation of metabolic process, intracellular signaling cascade, insulin signaling pathway and type 2 diabetes.

**Conclusion:**

Our study provides new insights into the role of miRNAs in the pathogenesis associated with nephrolithiasis.

## Introduction

Kidney stone disease (nephrolithiasis) remains a common health problem worldwide [1]. The exact formation mechanism of renal stones is complex and remains indistinct. Hyperoxaluria is a common finding in stone patients. The most common pathological condition involving oxalate is the formation of calcium oxalate stones in the kidney. Among all types of kidney stones, calcium oxalate monohydrate (COM) is the major crystalline compound in the stone formation (at a frequency of up to 77.5%) [2]. In addition to crystallization, crystal growth and crystal aggregation, the crucial mechanism for COM kidney stone formation is the adhesion of COM crystals to renal tubular epithelial cells [3], [4]. Adhesion of COM crystals can induce injury and apoptosis of renal epithelial cells. Meanwhile, COM-induced cellular injury can facilitate COM crystal adhesion [5], [6]. The vicious cycle therefore accelerates kidney stone formation. Understanding the alterations in renal tubular cells induced by COM crystals may lead to an identification of molecular targets for the prevention of kidney stone formation. However, changes in renal tubular epithelial cells under the influence of COM crystal-induced toxicity remain ambiguous.

MicroRNAs (miRNAs), a recently identified class of posttranscriptional gene regulators, may play an important role in COM crystal induced alteration of gene expression. MiRNAs are a group of small (20–22 nt) endogenous non-protein-coding RNA molecules that negatively regulate gene expression [9], [10]. These miRNAs usually bind to the 3′-untranslated region (3′-UTR) of target mRNA which leads to mRNA cleavage or translation inhibition [11]. It has also been predicted that miRNAs target more than 30% protein-coding genes [12]. However, there is no report on the effect of COM crystals on miRNAs in nephrolithiasis. Considering the potential roles of miRNAs in nephrolithiasis, we hypothesized that the cytotoxicity of COM crystals on HK-2 cells may be partially elicited by the regulation of miRNA expression levels. To our knowledge, this study presents the first miRNA analysis of human renal tubular cells injured by COM crystals. In this study, miRNA, mRNA microarray technology and quantitative real-time PCR (qRT-PCR) were used to investigate the effect of COM crystals exposure on the global expression profile of miRNAs in HK-2 cell line. We successfully identified some miRNAs that might help improve our understanding of the pathogenesis associated with stone formation, and more specifically, with the interactions between COM crystals and renal cells.

## Materials and Methods

### Cell Culture

Human Kidney Epithelial Cells, HK-2, were procured from American Type Culture Collection (ATCC) and maintained in a DMEM medium supplemented with 10% Fetal Bovine Serum and antibiotics. Before COM crystals treatment, cells were serum starved for 12 hours. Media components were procured from Invitrogen Corporation and all other chemicals were procured from Sigma-Aldrich.

### Preparation of COM Crystals

COM crystals were prepared by mixing equal volumes of 10 mM calcium chloride (CaCl_2_) and 10 mM sodium oxalate (Na_2_C_2_O_4_). The mixture was incubated overnight and COM crystals were harvested by centrifugation at 3000 rpm for 5 min. COM crystals were then decontaminated by UV light radiation for 30 min. These in vitro COM crystals had similar size and shape as those in vivo samples found in the urine of kidney stone patients.

### Cell Exposure

Prior to being used in experiments, COM crystals were resuspended to remove the aggregation between crystals. Confluent cells were treated with COM crystals at the concentration of 0, 0.1 mmol/L, 1 mmol/L and 10 mmol/L for 24 h. The media were collected for LDH assay, and then the cells were stained with DAPI. Cells treated with 1 mmol/L COM crystals for 24 h were used for miRNA and mRNA microarray.

### LDH Assay

Lactate dehydrogenase is a stable cytosolic enzyme which is released when there is injury on the cell membrane. The LDH which were released into the media was assessed by measuring LDH activity spectrophotometrically by lactate to pyruvate reaction. Reaction solutions contained collected media, 160 mM lactate, 25 mM NAD, 200 mM glycine-hydrazine buffer, PH 9.5. The absorbance at 340 nm was monitored for 60 min.

### Apoptosis Assay

HK-2 cells were grown in 6-well glass slides and when the cells were confluent. They were exposed to different concentrations of COM crystals (0, 0.1, 1, 10 mmol/L). The cells in the wells were washed with PBS, fixed with 1% formaldehyde for 10 min at room temperature, stained with 4′6-diamidino-2-phenylindole (DAPI) for two min and washed with PBS for 5 min. Cells were observed under the fluorescence microscope (Zeiss, Germany).

### RNA Isolation

HK-2 cells were treated with 1 mmol/L COM crystals, the samples were collected at 24 h after treatment. Cells treated with the same medium without COM crystals were used as the negative control. All samples were lysed in Trizol. RNA was extracted through the procedures recommended by the manufacture (Invitrogen, Carlsbad, USA) and DNA containment was removed through DNase digestion. RNA quality was confirmed by Agilent bioanalyzer 2100.

### miRNA Microarray and mRNA Microarray

The Agilent human miRNA microarrays (version 16.0) were used to compare the expression profile of control and COM crystals treated cells. 5 µg of total RNA was used for hybridization of miRNA microarray chip which contained 1205 human miRNAs (Shanghai Biochip Co.Ltd., China). The miRNA microarrays data discussed in this paper had been deposited in NCBI Gene Expression Omnibus and were accessible through GEO Series accession number GSE56934. mRNA expression profiles were analyzed using the Human lncRNA microarray v2.0 (Arraystar Company, USA). This microarray contained 30215 human mRNAs. The microarray data had been deposited in NCBI Gene Expression Omnibus and were accessible through GEO Series accession number GSE57111.

### Real-time RT-PCR

For miRNA detection, bulge-loop^tm^ miRNA qRT-PCR Primer Sets (one RT primer and a pair of qPCR primers for each set) specific for hsa-miR-638, has-miR-3125, hsa-miR-3195, hsa-miR-1260 and hsa-miR-371-5p, hsa-miR-933, and hsa-miR-4284 were designed by RiboBio (Guangzhou, China). For mRNA, total RNA was extracted and reversely transcribed to cDNA. PCR was carried out according to standard protocol of SYBR Premix Ex Taq with the aid of real-time PCR equipment. U6 and GAPDH were used as the negative control of miRNA and mRNA respectively. All the reactions were run in triplicate.

### Target Prediction and Function Analysis

The most dysregulated miRNAs after COM crystal exposure were selected for target prediction. TargetScan was used to predict the putative targets. The lists of potential gene targets for each selected miRNA were classified according to their biological functions which were determined by Gene Ontology system (http://www.geneontology.org/). To determine the possible overlapping of biological functions among these miRNAs, significantly overrepresented GO terms among all predicted gene targets for each individual miRNA were searched by means of the GOstat software. The program determined all the annotated GO terms associated with the target genes, and then counted the number of appearances of each GO term for these genes. A Fischer's exact test was then performed to give the *p*-value for each GO term, representing the probability that the observed counts could have been due to chance. In addition, Pathway analysis of the target genes was performed by using the DAVID Bioinformatics Resources 2008 (http://david.abcc.ncifcrf.gov/). The program groups together related the annotations (GO terms) for a similar set of genes, compared the GO processes if they might be related to a biological network and compiled a list of potential pathways for the effects of the target genes.

### Statistical Analysis

The continuous variables were expressed as the mean values±standard deviations (SD). Comparisons between the samples (control vs COM-exposed cells) were performed using one-way ANOVA followed by LSD's post hoc test. *P*<0.05 was considered statistically significant.

## Results

### LDH Release

**Figure 1:**
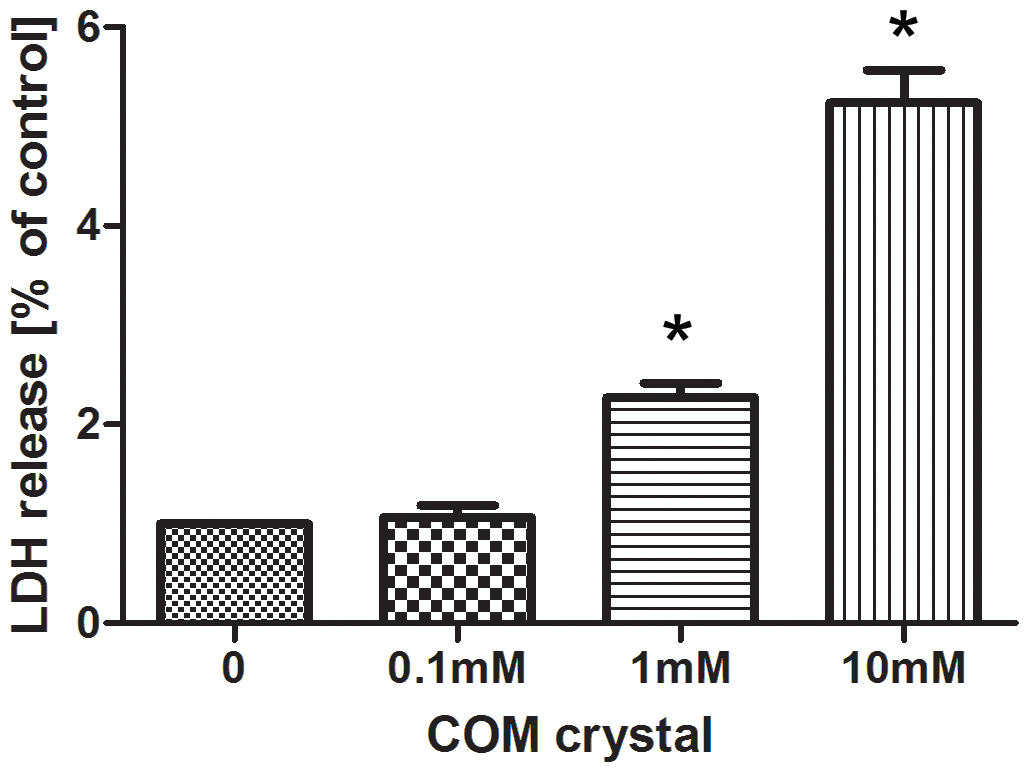
COM crystals increased LDH release from HK-2 cells. HK-2 cells were incubated with different concentrations (0, 0.1, 1, 10)mmol/L of COM for 24 h in triplicate samples to determine LDH activity released into media. Symbol (*) indicates significant difference from 0-control (*P*<0.05).

The release of LDH was measured as a marker for plasma membrane damage. Exposure to 1 mmol/L COM crystals for 24 h resulted in significantly higher levels of LDH released into media. Increased levels of LDH were measured in response to 10 mmol/L COM crystals (Figure 1). As expected, LDH release in response to COM crystals was concentration dependent.

### Apoptosis Assay

**Figure 2:**
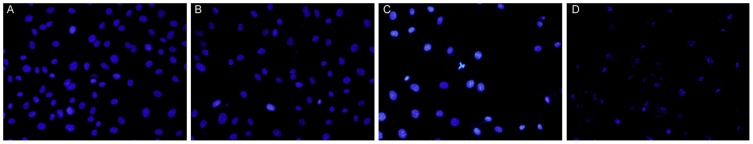
Cells in culture were examined by flurorescence microscopy following staining with DAPI. A Cells treated with 0(magnification, ×200).

COM crystals induced cytotoxicity in HK-2 cells. Condensed nuclei and apoptotic bodies were showed with DAPI staining after treatment with 1 mM COM crystals for 24 h (Figure 2). After treated with 10 mM COM crystals for 24 h, HK-2 cells showed highly condensed chromatin and many cell fragments. Based on the results of LDH release and apoptosis assay, we used 1 mM as the proper concentration for subsequent experiments.

### Alteration of miRNA Expression Profiles in HK-2 Cells after COM Crystals Treatment

Treatment with COM crystals significantly altered the miRNA expression profiles in HK-2 cells. We used the Agilent Human miRNA Microarray V16.0. This microarray has 1205 human miRNAs. 25 miRNAs were differentially expressed with a more than 1.5-fold change (Table 1). Among these miRNAs, 16 miRNAs were up-regulated, while 9 miRNAs were down-regulated.

**Table 1:** Dysregulated miRNAs after COM crystals exposure.

Probe_ID	foldchange	Probe_ID	Foldchange
Up-regulated		Down-regulated	
hsa-miR-371-5p	3.069941	hsa-miR-425*	0.069018
hsa-miR-3125	2.71054	hsv1-miR-H6-3p	0.071335
hsa-miR-638	2.682127	hsv1-miR-H7*	0.078529
hsa-miR-3195	2.251545	hsa-miR-933	0.09623093
ebv-miR-BART13	2.155447	hsa-miR-191*	0.303036
hsa-miR-135a*	2.078597	hsa-let-7f-1*	0.366442
hsa-miR-1274b	2.004063	hsa-miR-4284	0.393521
hsa-miR-497	1.918096	hsa-miR-4313	0.485941
hsa-miR-1181	1.829789	hsa-let-7b*	0.48814
hsa-miR-195	1.706755		
hsa-miR-1260b	1.693521		
hsa-miR-21*	1.688719		
hsa-miR-642b	1.644018		
hsa-miR-1260	1.585072		
hsv1-miR-H8	1.532887		
hsa-miR-1274a	1.505559		

miRNA cloning studies sometimes identify two ∼22 nt sequences miRNAs which originate from the same predicted precursor. When the relative abundancies clearly indicate which is the predominantly expressed miRNA, the mature sequences are assigned names of the form miR-56 (the predominant product) and miR-56* (from the opposite arm of the precursor). So the * means the product is from the opposite arm of the precursor. (http://www.mirbase.org/help/nomenclature.shtml).

**Figure 3:**
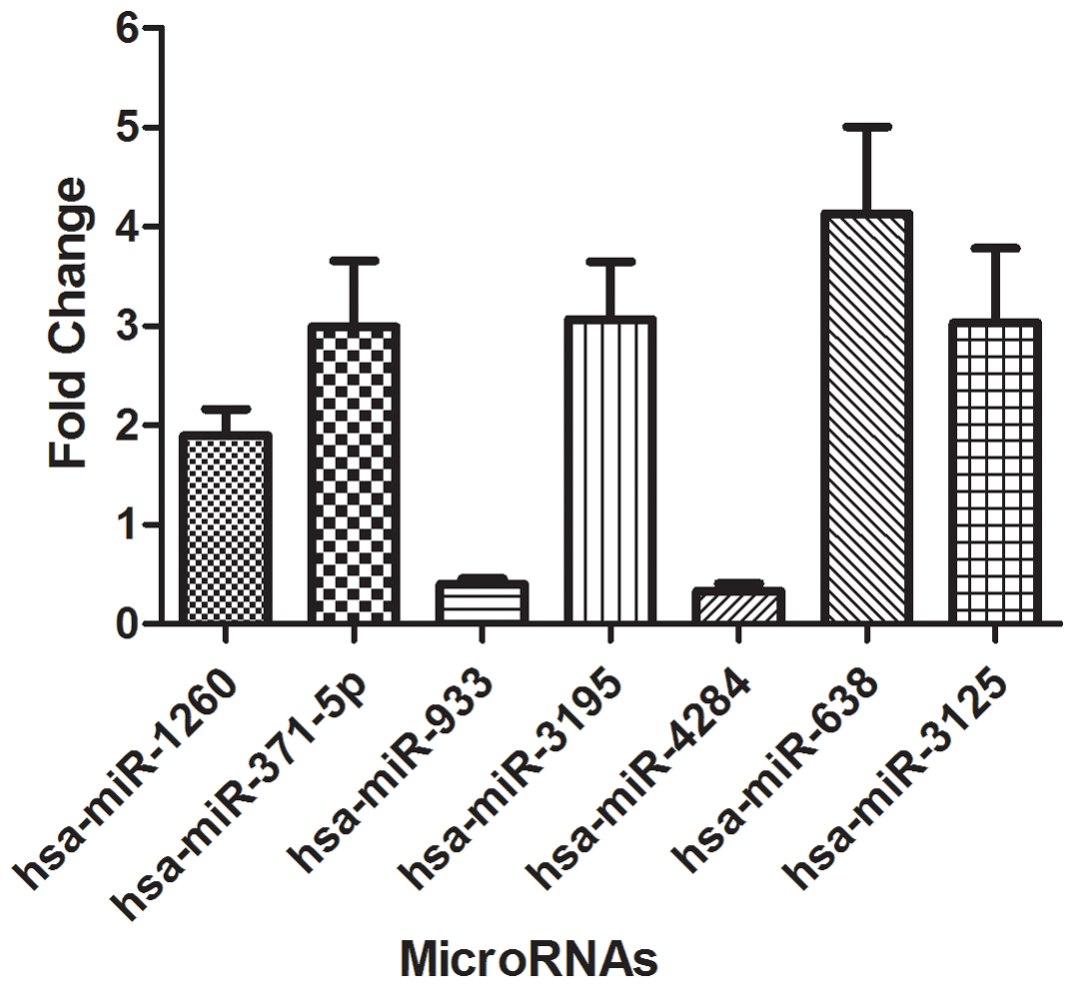
qRT-PCR was performed to confirm the expression of 7 selected miRNAs of HK-2 cells induced by COM crystals. The selected miRNAs included 5 up-regulated miRNAs and 2 down-regulated miRNAs.

To validate the data obtained from miRNA microarray, qRT-PCR was performed on seven differentially expressed miRNAs (five up-regulated: hsa-miR-638, has-miR-3125, hsa-miR-3195, hsa-miR-1260 and hsa-miR-371-5p; two down-regulated: hsa-miR-933, hsa-miR-4284) and the results from microarray and qRT-PCR were compared. As showed in Figure 3, hsa-miR-638, has-miR-3125, hsa-miR-3195, has-miR-1260 and has-miR-371-5p were up-regulated, while hsa-miR-933, hsa-miR-4284 were down-regulated according to the qRT-PCR data. The results were comparable with the microarray data and thus validated the results for miRNAs obtained from miRNA microarray.

**Figure 4:**
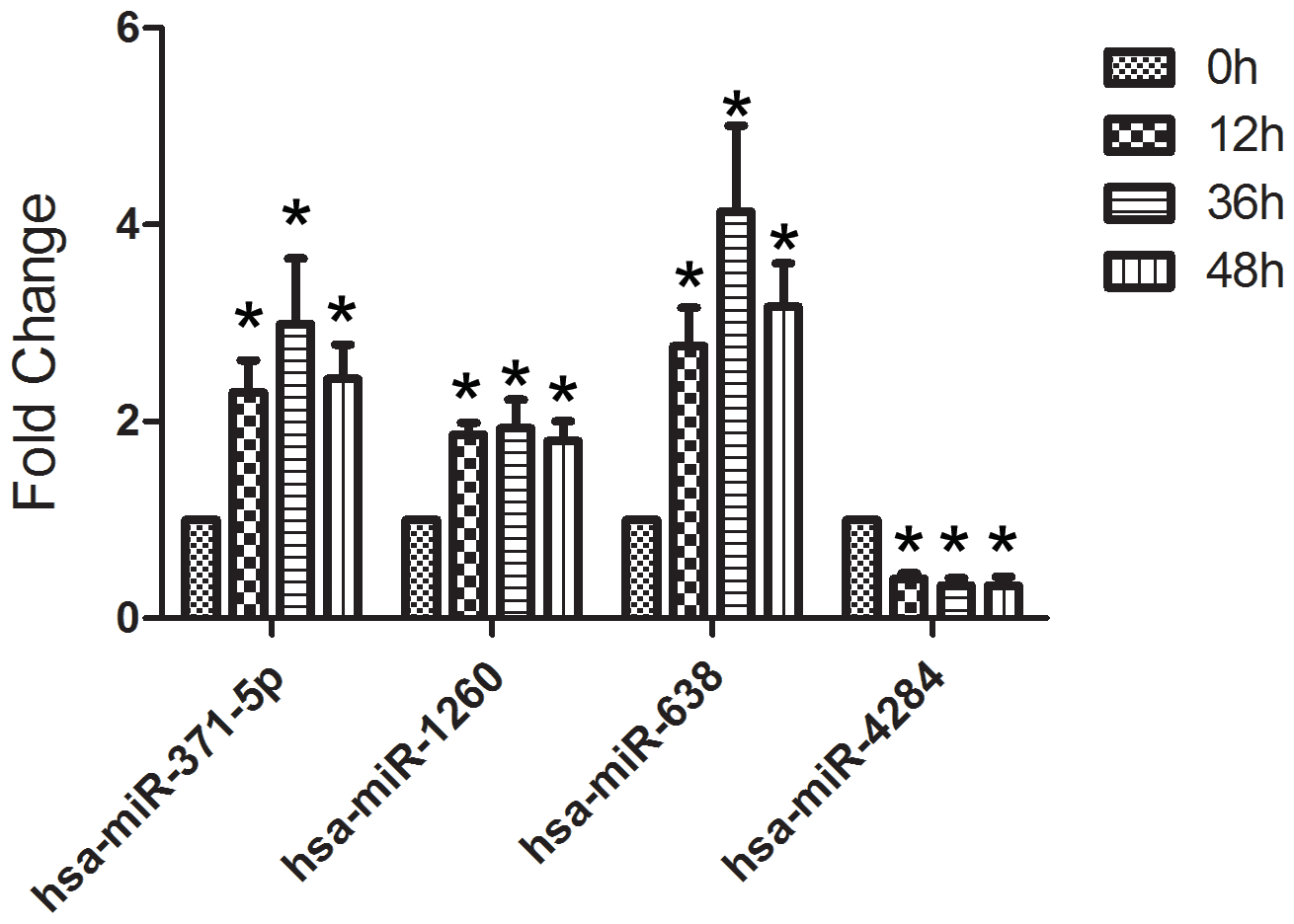
Expression profiles of 4 selected miRNAs of HK-2 cells induced by COM crystals with different time points.

To test the potential effect of different intervals of COM crystals on miRNA expression profiles, we selected four differentially expressed miRNAs (hsa-miR-638, hsa-miR-1260, hsa-miR-371-5p and hsa-miR-4284) to monitor their expression levels at 0 h, 12 h, 24 h and 48 h. Figure 4 showed a time-dependent regulation of miRNA expression in response to COM crystals treatment. hsa-miR-371-5p showed an initial increase at 12 h, which reached its top at 24 h after being exposed. At 48 h, the expression began to decrease compared with that at 24 h. The expression of hsa-miR-638 showed the same pattern of hsa-miR-371-5p. Besides, the expression of hsa-miR-1260 was almost the same at each time point (12 h, 24 h and 48 h). The expression of hsa-miR-4284 decreased sharply at 12 h, which reached its peak at 24 h. At 48 h, the expression of this miRNA was almost the same compared with that at 24 h.

### Identification of mRNA Expression Profiles in HK-2 Cells after COM Crystals Treatment

To explore the impact of gene expression, we further studied the mRNA expression patterns of HK-2 cells treated with COM crystals by using the human lncRNA microarray v2.0 (Arraystar Company, USA). The microarray has 30215 human mRNAs. In total, 2592 genes were identified as differentially expressed (Table 2). Among these genes, 1764 were up-regulated, while 828 were down-regulated. 11 genes were differentially up-regulated with a more than 16-fold change. The top 10 up-regulated genes were IRX6, CLIP1, MMP25, CCL3, C10orf107, PLEC, CCL3L1, CCL3L3, TNF and KRTAP11-1. 87 genes were differentially down-regulated with a more than 16-fold change. The top 10 down-regulated genes were CTDSP2, QRICH2, NINL, USP37, C8orf86, PPEF1, KIAA1310, TRIM36, FDPS, RAPH1. (Table 3)

**Table 2:** Summary of data from mRNA microarray.

			mRNAs		
	Fold change 2–4	Fold change 4–16	Fold change>16	Total	Changed mRNAs
Up-regulation	1361	392	11	1764	2592
down-regulation	466	275	**87**	**828**	

**Table 3:** Dysregulated mRNAs after COM crystals exposure.

Up-regulated		Down-regulated	
mRNAs	Log2 Fold change (T/C)	mRNAs	Log2 Fold change (T/C)
IRX6	8.485357	CTDSP2	-10.556264
CLIP1	5.7348537	QRICH2	-8.124645
MMP25	5.0595646	NINL	-7.7819004
CCL3	4.8733883	USP37	-7.639219
C10orf107	4.7241697	C8orf86	-6.735654
PLEC	4.6747723	PPEF1	-6.5349064
CCL3L1	4.6711597	KIAA1310	-5.998905
CCL3L3	4.585658	TRIM36	-5.6827116
TNF	4.5506287	FDPS	-5.438601
KRTAP11-1	4.447587	RAPH1	-5.268367

**Figure 5:**
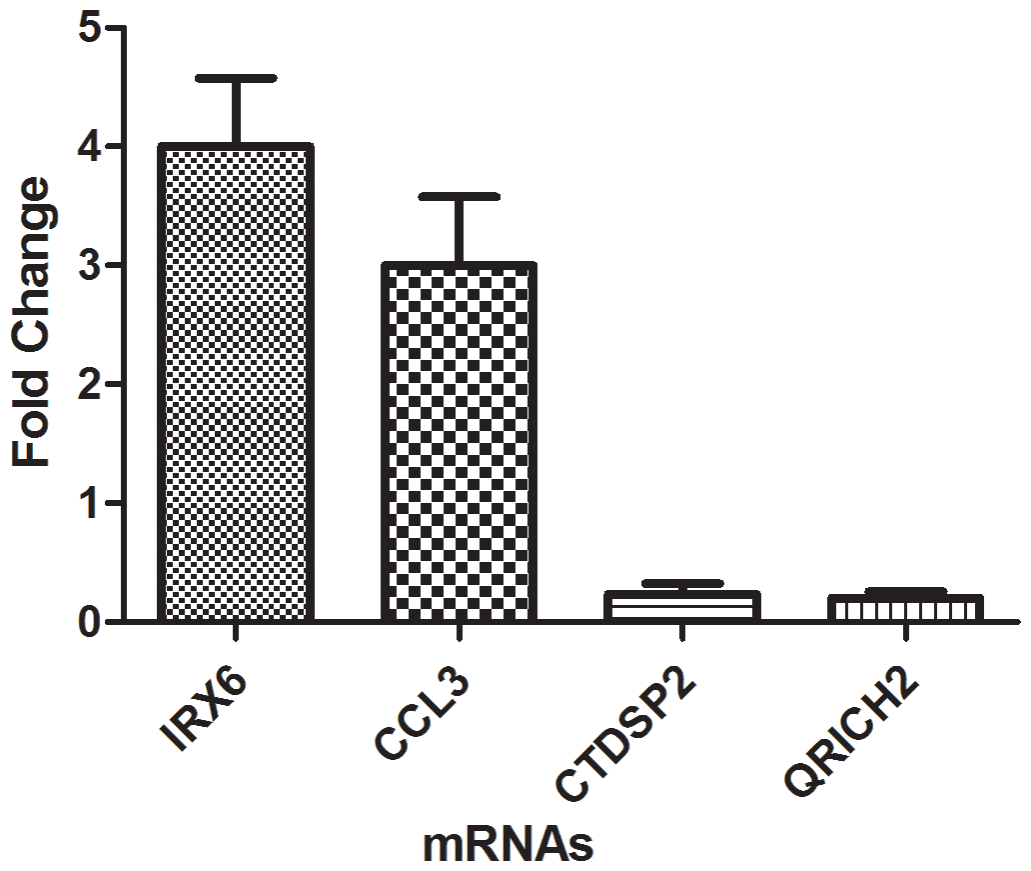
qRT-PCR was performed to confirm the expression of 4 selected mRNAs of HK-2 cells induced by COM crystals. The selected mRNAs included 2 up-regulated mRNAs and 2 down-regulated mRNAs.

To validate the microarray data, four differentially expressed genes were randomly chosen for qRT-PCR (two up-regulated: IRX6, CCL3; two down-regulated: CTDSP2, QRICH2) (Figure 5). Our results were comparable with the microarray data and thus validated the results for mRNAs obtained from mRNA microarray.

### Integrated Analysis of Deregulated miRNAs and mRNAs

MiRNAs modulate gene expression through mRNA degradation or translation repression. Therefore, we performed an integrated analysis of miRNA and mRNA expression patterns. The computational program, TargetScan, was utilized to predict the target genes. These genes were compared with those from mRNA microarray. Among the 2592 differentially expressed mRNAs, 114 genes were the predicted targets of the 12 differential miRNAs (Table 4). Some of the genes were related to cell death, mitochondrion and metabolic process.

**Table 4:** The genes both contained in mRNA chips and miRNAs prediction.

Gene name	Description
hsa-miR-933	NR4A1 MBP PLXNA1 C19orf25 MID1IP1
hsa-miR-642b	SEMA3A PRDM4 C9orf91 AK3L1 ENO2 CIDEA
hsa-miR-638	CHRNB4 EXOC7 GPX1 SMAP2 CHAC1 CPNE1 CHD3 CAPN10 ENO2 CNPY4 PLXNA1 LMBR1L
hsa-miR-497	SEMA3A SPON2 PRDM4 SFRS16 CHRNB4 CPXM2 CMTM3 TMEM174 FAM122B C11orf42 C9orf91 AK3L1 EXT2 PUF60 EXOC7 FUT3 CCDC19 FAM32A GLS2 PDCD4 SLC13A5 CYB561D1 RSPO1 GPX1 THYN1 GSR HMOX2 NR4A1 C1QL4 APP ITIH1 KARS KRT33B LDHA MAGEB4 MBP ASB1 CMPK1 PGM1 AURKAIP1 PPP1R2 PRKAR2A C12orf4 USP31 TGIF2 RFWD2 GORASP1 SMAP2 MRPL40 SNRPA1 SSRP1 TTC1 ZYX CHAC1 C13orf18 POLDIP3 CUL2 SLITRK2 SPRYD3 NAE1 EIF2S2 CPNE1 CCNE1 C1orf38 NCOR2 KIAA0430 DEPDC5 BZW1 SCRN1 CDC25A TSGA13
hsa-miR-4313	SEMA3A PRDM4 CMTM3 C11orf42 C9orf91 PDCD4 CYB561D1 RSPO1 THYN1 HMOX2 MBP CMPK1 TGIF2 GORASP1 ZYX POLDIP3 SPRYD3 CCNE1 NCOR2 SCRN1 CDC25A TSGA13 CCT3 CLPTM1L EI24 C19orf25 MID1IP1 ZNF384 GPR114 B4GALT2 TMEM44 PDIA4 C1orf162 CIDEA
hsa-miR-4284	CHRNB4 CPXM2 CMTM3 FAM122B CYB561D1 RSPO1 GSR TGIF2 RFWD2 C13orf18 POLDIP3 CUL2 DEPDC5 BZW1 LMBR1L CCR6 NDUFV3 CCT3 FOXM1 C1orf162 CIDEA
hsa-miR-371-5p	SEMA3A FAM122B C9orf91 AK3L1 SLC13A5 RSPO1 GSR LDHA MBP ASB1 CMPK1 PPP1R2 C13orf18 CUL2 KIAA0430 BZW1 SCRN1 CDC25A CCR6 SGSM3 NDUFV3 NEK3 SUFU RPL37A CCT3 CLPTM1L GFI1B EI24
hsa-miR-3663-3p	SPON2 CPXM2 CMTM3 C1QL4 CMPK1 SMAP2 MPPED1 CHAC1 C13orf18 POLDIP3 EIF2S2 NCOR2 CDC25A CNPY4 PLXNA1 CCR6 NDUFV3 ZNF384 GPR114 FOXM1
hsa-miR-3125	PRDM4 CPXM2 C9orf91 EXT2 PUF60 FAM32A GLS2 GPX1 GSR KRT33B MBP CMPK1 PGM1 PPP1R2 PRKAR2A C12orf4 SMAP2 TTC1 SLITRK2 SPRYD3 KIAA0430 CHD3 ENO2 CNPY4 CCR6 SUFU RPL37A CLPTM1L GFI1B ZNF384 DTNB FOXM1 C1orf162
hsa-miR-195	SEMA3A SPON2 PRDM4 SFRS16 CHRNB4 CPXM2 CMTM3 TMEM174 FAM122B C11orf42 C9orf91 AK3L1 EXT2 PUF60 EXOC7 FUT3 CCDC19 FAM32A GLS2 PDCD4 SLC13A5 CYB561D1 RSPO1 GPX1 THYN1 GSR HMOX2 NR4A1 C1QL4 APP ITIH1 KARS KRT33B LDHA MAGEB4 MBP ASB1 CMPK1 PGM1 AURKAIP1 PPP1R2 PRKAR2A C12orf4 USP31 TGIF2 RFWD2 GORASP1 SMAP2 MRPL40 SNRPA1 SSRP1 TTC1 MPPED1 ZYX CHAC1 C13orf18 POLDIP3 CUL2 SLITRK2 SPRYD3 NAE1 EIF2S2 CPNE1 CCNE1 C1orf38 NCOR2 KIAA0430 DEPDC5 BZW1 SCRN1 CDC25A
hsa-miR-1260b	SEMA3A PRDM4 TMEM174 FAM122B AK3L1 EXT2 EXOC7 FUT3 GLS2 PDCD4 SLC13A5 CYB561D1 NR4A1 APP MAGEB4 ASB1 PPP1R2 PRKAR2A C12orf4 USP31 TGIF2 GORASP1 SMAP2 SSRP1 MPPED1 CUL2 EIF2S2 C1orf38 NCOR2 DEPDC5 BZW1 SCRN1 CDC25A CHD3 CAPN10 ENO2 CNPY4 LMBR1L CCR6 SGSM3 NDUFV3 NEK3 SUFU GFI1B EI24 C19orf25 MID1IP1 OTOA ZNF384 DTNB GPR114 FOXM1 GPX4 NT5C SKAP1 B4GALT2 TMEM44 PDIA4
hsa-miR-1260	SEMA3A PRDM4 TMEM174 FAM122B AK3L1 EXT2 EXOC7 FUT3 GLS2 PDCD4 SLC13A5 CYB561D1 NR4A1 APP MAGEB4 ASB1 PPP1R2 PRKAR2A C12orf4 USP31 TGIF2 GORASP1 SMAP2 SSRP1 MPPED1 CUL2 EIF2S2 C1orf38 NCOR2 DEPDC5 BZW1 SCRN1 CDC25A CHD3 CAPN10 ENO2 CNPY4 LMBR1L CCR6 SGSM3 NDUFV3 NEK3 SUFU GFI1B EI24 C19orf25 MID1IP1 OTOA ZNF384 DTNB GPR114 FOXM1 GPX4 NT5C SKAP1 B4GALT2 TMEM44 PDIA4

The basic biological function of each putative gene was classified by means of the Gene Ontology system. Since every single gene was associated with many GO terms, the GOsat software was used to identify the overrepresented GO terms for each miRNA. Table 5 presents a few representative biological processes associated with each miRNA as predicted by the GOstat software. The putative targets of these miRNAs were then used for the pathway analysis performed by DAVID Bioinformatics Resources. The program provided potential functional pathways for the putative target genes of the selected miRNAs. Based on the target analysis, we found several important biological processes, such as apoptosis, regulation of RNA metabolic process, intracellular signaling cascade, regulation of kinase activity, I-kappaB kinase/NF-kappaB cascade, mitochondrion, response to wounding, regulation of transcription and ion binding transcription. These results suggested important roles these miRNAs played in human health and disease regulations. Furthermore, the pathway analysis suggested an important regulatory role these miRNAs played in different biological processes such as insulin signaling pathway, type 2 diabetes mellitus, porphyrin and chlorophyll metabolism (Table 5 and Table 6).

**Table 5:** Analysis of biological processes of the predicted miRNAs targets by GOstat.

miRNAs	GO process	Target genes	Count	%	*P*-value
hsa-miR-497	GO:0043549:regulation of kinase activity	PPP2R1A, MADD, PTPLAD1, DRD5, PDCD4, CDC25A, CDC25B, CBLC, TARBP2, PRKAR2A, APP, TSPYL2, DUSP9, HTR2A	14	0.3688	0.04444
	GO:0007249:I-kappaB kinase/NF-kappaB cascade	PTPLAD1, SNIP1, TLR1, IKBKB, TNIP2	5	0.1317	0.04600
	GO:0031980:mitochondrial lumen	GLS2, MRPL40, PDPR, FECH, HARS2, TMLHE, CARS2, YARS2, AK3L1, LRPPRC, KARS	11	0.2898	0.02395
	GO:0005739:mitochondrion	MRPL40, ACOX1, D2HGDH, CCDC19, NLRX1, AK3L1, KCNJ11, KARS, GLS2, GPX1, GSR, HTRA2, CISD3, SLC25A22, HARS2, SLC25A2, BDH2, AATK, PDPR, PPP2R1A, TRMU, FECH, NOL3, MRPS2, MMAB, RAB40AL, UCP2, TMLHE, SLC25A35, CARS2, YARS2, ALKBH3, LRPPRC	33	0.8693	0.03916
hsa-miR-638	GO:0009611:response to wounding	CCL11, MYF6, GPX1, TNFRSF1B, F10, MGLL, HOXB13, IL11	8	0.9335	0.00599
	GO:0010740:positive regulation of protein kinase cascade	GPX1, F10, BST2, IL11	4	0.4667	0.03180
	GO:0031099:regeneration	MYF6, GPX1, NR4A3	3	0.3501	0.03308
hsa-miR-1260	GO:0051252:regulation of RNA metabolic process	ZNF583, SNIP1, PDX1, ZNF253, SKAP1, IL11, APP, GFI1B, RAB26, SLA2, OTX1, ARID5A, SOX12, HDAC10, FOSB, NPAS4, SPEN, UBN1, ZNF192, ZNF786, MED8, SERBP1, TGIF2, CUX1, TFAP2E, MAPRE3, MED1, CARHSP1, FOXM1, HOXB13, FHL2, ZNF367, NEDD8, ZNF177, SUFU, ZNF615, ZNF169, TNFRSF1B, TSC22D2, TCEA3, HEXIM2, LHX1, OVOL1, GATAD2B, ZNF426, ZNF701, FOXD4, SIM2, CHD3, MYF6, ESRRA, ZNF565, CREBZF, TAF8, NR4A1, ZNF221, NR4A3, ZNF763, RFX3, MESP2, NCOR2	61	1.5204	0.00323
	GO:0045449:regulation of transcription	ZNF583, SNIP1, PDX1, ZNF253, PDCD4, CBX7, SKAP1, IL11, ARHGAP22, BZW1, APP, LBH, GFI1B, MED29, RAB26, SSBP2, POGZ, OTX1, SLA2, SOX12, ARID5A, HDAC10, FOSB, HDAC11, SPEN, NPAS4, UBN1, CCNL2, ZNF192, CCR6, PRDM4, ZNF786, MED8, PARP14, ZNF384, ERN2, TGIF2, CUX1, TFAP2E, MAPRE3, MED1, CARHSP1, FOXM1, TRIB3, HOXB13, FHL2, ZNF367, NEDD8, ZNF177, ZNF615, SUFU, ZNF169, TSC22D2, TCEA3, HEXIM2, LHX1, OVOL1, GATAD2B, ZNF426, RNF10, ZNF701, FOXD4, CHD3, SIM2, MYF6, SSRP1, ESRRA, ZBTB46, ZNF565, CREBZF, TAF8, NR4A1, ZNF221, NR4A3, ZNF629, PHF19, ZNF763, RFX3, IKBKB, MESP2, NCOR2	80	1.9940	0.00686
	GO:0043167:ion binding	SLC13A5, ZNF583, ZNRF1, ZNF253, TTL, APOBEC3C, APOBEC3D, MAP3K5, APP, CISD3, CDH23, RET, CAPNS2, FECH, PDXK, CRTAC1, BSN, RNASEH2A, WDR92, ZNF192, PRDM4, ZNF786, EIF2S2, ZNF384, UNC13A, NEK3, ZNF615, KCNJ1, RPS27, ZNF428, LHX1, OVOL1, RNF168, RNF10, ZNF426, STK19, GABRP, GIT1, NPLOC4, ESRRA, ZBTB46, B4GALT2, SETDB2, NR4A1, ZNF221, NR4A3, ACACB, ATP13A3, CYB561, PRPSAP2, ZNF629, TRIM56, KCNJ9, TMLHE, ZNF385D, SPTA1, TRAFD1, GLRA1, MLPH, GLRA3, CYP11B2, PDIA4, KCNJ11, KCNIP1, USP19, SMAP2, GFI1B, STAC2, TRIM9, DBR1, NSF, NT5C, POGZ, MICAL3, RPH3AL, CYB5B, ARL3, MYRIP, MAP3K15, ZNF691, TAF15, ERN2, CA4, CLCNKA, ZNF367, FHL2, EGLN2, ZNF177, BEST4, ZNF169, CYB561D1, TCEA3, CALML3, MMACHC, PRRG2, RASGRP2, DTNB, ENO2, MT1E, GATAD2B, GALNT11, ZNF701, CHD3, MT1M, NOS1, ZNF565, TRIM29, TRIM25, CYP4F8, RPS6KA3, FBLN1, RASSF5, PHF19, CDH15, ARSA, SCN4B, SUMF2, ZNF763, ALKBH3, RNF40	120	2.9910	0.03602
hsa-miR-1260b	GO:0051252:regulation of RNA metabolic process	ZNF583, SNIP1, PDX1, ZNF253, SKAP1, IL11, APP, GFI1B, RAB26, SLA2, OTX1, ARID5A, SOX12, HDAC10, FOSB, NPAS4, SPEN, UBN1, ZNF192, ZNF786, MED8, SERBP1, TGIF2, CUX1, TFAP2E, MAPRE3, MED1, CARHSP1, FOXM1, HOXB13, FHL2, ZNF367, NEDD8, ZNF177, SUFU, ZNF615, ZNF169, TNFRSF1B, TSC22D2, TCEA3, HEXIM2, LHX1, OVOL1, GATAD2B, ZNF426, ZNF701, FOXD4, SIM2, CHD3, MYF6, ESRRA, ZNF565, CREBZF, TAF8, NR4A1, ZNF221, NR4A3, ZNF763, RFX3, MESP2, NCOR2	61	1.5204	0.00323
	GO:0007242:intracellular signaling cascade	FGFR1, CARHSP1, RAB9A, RAB3D, PTPLAD1, DRD5, SNIP1, MAPKAPK3, FHL2, EGLN2, PDX1, RAB42, MAP3K5, DIRAS1, PRKAR2A, KISS1R, STAC2, RASGRP2, RAB26, TNIP2, LTB, GDI1, RET, C5AR1, PIK3C2B, SLA2, STMN4, NFKBIL1, DEPDC5, ARL3, RASL11B, RASSF5, RPS6KA3, RAB37, CNIH3, BBC3, SGSM3, RAB17, ASB1, IKBKB, UNC13A, MED1	42	1.0469	0.01837
hsa-miR-3125	GO:0006350:transcription	RNASEK, FOXM1, CBX4, TTLL5, ZNF367, ZNF177, CBX7, ZNF615, DPF1, ZNF169, GFI1B, OVOL1, GATAD2B, TWIST2, FOXD4, CHD3, ZNF548, ZBTB46, BRF2, TRIM29, SOX12, CDK9, DACH1, NR4A3, SPEN, HDAC11, HMGA1, NRIP2, CCR6, PRDM4, MED8, PARP14, ZNF384, RFX3, LRPPRC, PUF60, MED1	37	1.9004	0.01354
hsa-miR-3195	GO:0002064:epithelial cell development	AGPAT6, HOXB13	2	0.9009	0.02456
	GO:0004672:protein kinase activity	FGFR1, LMTK2, CDK9, IKBKB	4	1.8018	0.04223
hsa-miR-4284	GO:0008629:induction of apoptosis by intracellular signals	CUL3, CUL2, BBC3, CIDEA	4	0.1980	0.02512
hsa-miR-4313	GO:0045449:regulation of transcription	CARHSP1, ZNF583, GDF7, HOXB13, CBX7, PDCD4, IL11, DPF1, CCNE1, TSPYL2, MED29, OVOL1, TWIST2, ESRRA, ZBTB46, POGZ, BRF2, ARID5A, HDAC11, DACH1, ZNF497, HMGA1, CCNL2, ZNF629, TARBP2, PRDM4, MED8, PARP14, ZNF384, ZNF763, TGIF2, JAK3, CUX1, MAPRE3, MESP2, LRPPRC, NCOR2	37	2.2534	0.02350

**Table 6:** Pathway analysis of the predicted miRNAs using DAVID Bioinformatics software.

Category	Term	Count	%	*P*-value	Genes
hsa-miR-497 KEGG-PATHWAY	hsa04910:Insulin signaling pathway	9	0.237092	0.00939	CBLC, PRKAR2A, EXOC7, SLC2A4, FASN, ACACB, PIK3R3, IKBKB, LIPE
hsa-miR-195 KEGG_PATHWAY	hsa04910:Insulin signaling pathway	9	0.2426530	0.00802	CBLC, PRKAR2A, EXOC7, SLC2A4, FASN, ACACB, PIK3R3, IKBKB, LIPE
hsa-miR-1260b KEGG-PATHWAY	hsa04930:Type II diabetes mellitus	5	0.124626	0.02158	SLC2A4, PDX1, PIK3R3, IKBKB, KCNJ11
hsa-miR-3195 KEGG-PATHWAY	hsa04910:Insulin signaling pathway	3	1.351351	0.00405	CALML3, FASN, IKBKB
hsa-miR-4313 KEGG-PATHWAY	hsa00860:Porphyrin and chlorophyll metabolism	3	0.182704	0.03826	HMOX2, ALAD, BLVRB

## Comment

Nephrolithiasis is a multi-factorial disorder which, in many patients, results in renal deposition of calcium oxalate. There are various theories in the pathogenesis of nephrolithiasis. One favored theory proposes that oxalate-induced injury to renal tubular epithelial cells promotes adherence of calcium oxalate crystals. The renal damage is closely linked to the degree of COM crystals accumulation in the kidney and most likely results from a COM-induced injury to proximal tubular cells. Therefore, we used HK-2 cells exposure to COM crystals to simulate the process of renal damage.

In our study, the first step demonstrated that COM crystals had concentration-dependent toxicity on HK-2 cells. Based on these results, we found a proper concentration for our subsequent experiments.

HK-2 cells are a line of human proximal tubular epithelial cells immortalized by using the E6/E7 genes of human papilloma virus (HPV 16) [7]. These cells retain the characteristics of proximal renal tubular epithelium and have been successfully used as an in vitro model system to represent the human kidney epithelial cells. HK-2 cells, instead of other cells, were used in this study because the proximal tubule is the major site for renal oxalate handing [8]. Moreover, HK-2 cell line has been frequently used in several previous studies on COM crystal-induced renal tubular cell injury [13], [14], [15]. However, HK-2 cells are not normal proximal tubular cells, which is also one limitation of the present study. In our future study, we will focus on normal proximal tubular cells.

Over the past two decades, many studies [16], [17] have demonstrated that calcium oxalate interactions with renal epithelial cells result in a program of events, including changes in gene expression and cell dysfunction. Calcium oxalate toxicity will induce new gene expression and protein synthesis. Besides, the renal epithelial cells exposed to oxalate must be able to adapt to oxalate stress. Many signaling pathways, including p38 MAPK and JNK, are activated in renal tubular epithelium in response to oxalate and COM crystals [16], [18]. However, previous studies have mainly focused on DNA, mRNA and protein levels. Koul et al utilized gene chips to explain the global gene expression changes in human renal epithelial cells after exposure to oxalate. Their results showed that renal cells exposed to oxalate resulted in the regulation of genes that were associated with specific molecular function, biological processes, and other cellular components [13]. In proteomic insights, chen et al identified 12 differentially expressed proteins in HK-2 cells induced by oxalate and COM crystals. These proteins were associated with cell propagation and apoptosis, protein synthesis and cellular energy metabolism [14]. In another study, 53 proteins were altered in MDCK cells after exposure to high-dose COM crystals for 48 h. These proteins were involved in protein biosynthesis, ATP synthesis, cell cycle regulator, cellular structure and signal transduction [19]. Besides these mechanisms, miRNA is a newly identified mechanism, and this field is potentially promising. Thus far, several studies have investigated miRNA expression patterns in prostate and renal cancers in urology [20], [21], [22]. To explore this new field in stone formation, we examined the miRNA expression profiles of HK-2 cells treated with COM crystals, and identified some deregulated miRNAs. In this study, we dentified some deregulated miRNAs which were involved in biological processes, apoptosis, intracellular signaling cascade, protein kinase activity, etc. The results were confirmed by qRT-PCR.

To study the involvement of miRNA-mediated regulation of gene expression in HK-2 cells, we analyzed whether the genes from mRNA microarray were the predicted targets of the deregulated miRNAs, and finally, the results showed that 114 genes were the predicted targets of the 12 differential miRNAs. hsa-miR-642b, hsa-miR-4313 and hsa-miR-4284 were related to cell death, mitochondrion and metabolic process; hsa-miR-497, hsa-miR-4313 and hsa-miR-195 were related to programmed cell death; hsa-miR-497, hsa-miR-195 and hsa-miR-371-5p were related to lactate dehydrogenase; hsa-miR-195 was related to mitochondrial function; hsa-miR-497, hsa-miR-3125, hsa-miR-1260b and hsa-miR-195 were related to protein kinase.

According to the GO analysis, hsa-miR-497 and hsa-miR-3195 may be involved in the regulation of protein kinase activity; hsa-miR-1260b may be involved in intracellular signaling cascade; hsa-miR-1260 and hsa-miR-1260b may be involved in the regulation of RNA metabolic process; hsa-miR-4313 and hsa-miR-1260 may be involved in the regulation of transcription; hsa-miR-497 may be involved in I-kappaB kinase/NF-kappaB cascade; hsa-miR-4284 may be involved in apoptosis by intracellular signals; hsa-miR-638 may be involved in response to wounding; hsa-miR-497 may be involved in mitochondrion function. In previous reports, MAPK signaling pathways and NF-kappaB were always related to the COM crystals-cell interaction. Peerapen et al indicated that COM crystals caused tight junction disruption in distal renal tubular epithelial cells through p38 MAPK activation [23]. Chaturvedi et al found that oxalate exposure resulted in re-initiation of the DNA synthesis, altered gene expression and apoptosis. Exposure to oxalate rapidly stimulated robust phosphorylation and activation of p38 MAPK. Oxalate exposure induced modest activation of c-Jun. p38 MAPK activity was essential for the effects of oxalate on re-initiation of DNA synthesis [24]. Oxalate inhibited renal proximal tubule cell proliferation via oxidative stress and p38 MAPK/JNK signaling pathways [25]. Tozawa et al demonstrated that oxalate induced OPN expression by activating NF-kappaB in renal tubular cells [26]. Koul et al suggested that the interaction of COM crystals with renal cells had been proved to result in altered gene expression and re-initiation of DNA synthesis [27]. Another study indicated that the exposure to high levels of COM crystals was injurious to renal epithelial cells [28]. Under transmission electron microscopy, proximal tubular cells were capable of internalizing COM crystals since COM could interact with mitochondria directly. Treatment with COM crystals induced a significant decrease in the mitochondrial membrane potential [29]. Our studies found that the genes involved in MAPK signaling pathways, NF-kappaB and mitochondrial membrane potential were also regulated by miRNAs as mentioned before.

Pathway analysis based on the differentially expressed mRNAs revealed significant pathways with *P*-values<0.05, including the insulin signaling pathway, type 2 diabetes mellitus, porphyrin and chlorophyll metabolism pathway. In a preceding study, kidney stone disease was related to the history of T2DM (OR: 2.44; 95% CI, 1.84–3.25) and that of insulin use (OR: 3.31; 95% CI, 2.02–5.45). They concluded that the history of T2DM, the use of insulin, FPI and HbA_1_c remained significantly associated with kidney stone disease [30]. In a 5-year follow-up, patients who received a diagnosis of urinary calculi were at an increased risk for diabetes mellitus [31]. Some studies demonstrated an increase in urinary calcium and phosphorus excretion in patients with T2DM [32]. Recent investigations had highlighted that stone formers with T2DM had increased urinary oxalate excretion [33]. The mechanism between stone disease and diabetes is still unknown, but recent literature has indicated that the production of reactive oxygen species (ROS) and development of oxidative stress (OS) might be in common pathways [34]. In our studies, pathway analysis indicated miRNAs mentioned previously might be involved in the insulin signaling pathway and type 2 diabetes mellitus. However, a lot of works still need to be done to validate the connection between stone disease and diabetes.

It should be noted that we evaluated changes in miRNA in HK-2 cells after 24 h exposure to 1 mmol/L of COM crystals. Although we successfully identified several miRNAs that were altered in this setting, the evaluation of changes caused by higher doses of COM crystals and at later time points would lead to an understanding of “late responses” in proximal renal tubular cells. Moreover, the study of miRNA changes in distal tubular cells would provide more insights into the molecular mechanisms of COM crystal-induced toxicity.

## Conclusions

In summary, we successfully identified a set of miRNAs in HK-2 cells that were altered by COM crystal-induced toxicity. Alterations in several of these miRNAs could help us further explore the mechanistic pathways of COM crystal-induced toxicity in renal tubular epithelial cells. However, the functional significance of some altered miRNAs remains unclear and should be further elucidated in order to gain a better understanding of the detrimental changes and the adaptive responses in these cells during COM crystal adhesion, and finally to unravel the pathogenic mechanisms of kidney stone disease.
